# Electrically Induced Liquid Metal Droplet Bouncing

**DOI:** 10.1021/acs.langmuir.2c00577

**Published:** 2022-05-26

**Authors:** Shubhi Bansal, Yutaka Tokuda, Jonathon Peasley, Sriram Subramanian

**Affiliations:** † University College London, London WC1E 6BT, U.K.; ‡ City University of Hong Kong, Kowloon 518057, Hong Kong, China; § University of Sussex, Brighton BN1 9RH, U.K.

## Abstract

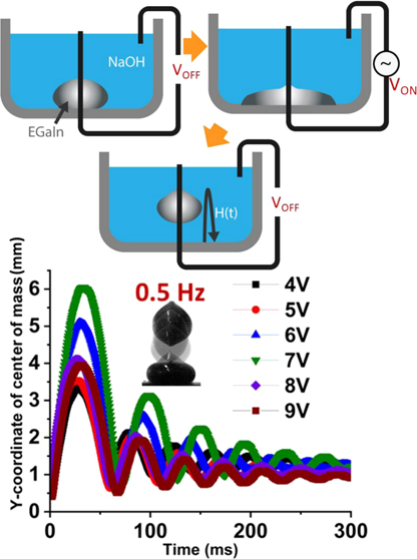

Liquid metals, including eutectic gallium–indium (EGaIn), have been explored for various planar droplet operations, including droplet splitting and merging, promoting their use in emerging areas such as flexible electronics and soft robotics. However, three-dimensional (3D) droplet operations, including droplet bouncing, have mostly been limited to nonmetallic liquids or aqueous solutions. This is the first study of liquid metal droplet bouncing using continuous AC electrowetting through an analytical model, computational fluid dynamics simulation, and empirical validation to the best of our knowledge. We achieved liquid metal droplet bouncing with a height greater than 5 mm with an actuation voltage of less than 10 V and a frequency of less than 5 Hz. We compared the bouncing trajectories of the liquid metal droplet for different actuation parameters. We found that the jumping height of the droplet increases as the frequency of the applied AC voltage decreases and its amplitude increases until the onset of instability. Furthermore, we model the attenuation dynamics of consecutive bouncing cycles of the underdamped droplet bouncing system. This study embarks on controlling liquid metal droplet bouncing electrically, thereby opening a plethora of new opportunities utilizing 3D liquid metal droplet operations for numerous applications such as energy harvesting, heat transfer, and radio frequency (RF) switching.

## Introduction

Room temperature liquid metals have gained significant attention with their numerous applications for soft robotics and flexible electronics such as micro-electro-mechanical system (MEMS) switches,^1^ haptic and visual displays,^2,3^ reconfigurable radio frequency (RF) systems,^4^ and many others.^5^ Liquid metals have unique properties of low viscosity, high thermal and electrical conductivity, and large surface tension, which support the active manipulation of droplets using an electric field.^6^ The electrically induced liquid oscillations for conductive and dielectric nonmetallic liquid droplets have been researched for multiple decades;^7−9^ however, effective electrical manipulation of liquid metals originated with the discovery of continuous electrowetting (CEW).^10^ Electrowetting has been explored with various liquid metals,^11^ including aluminum,^12^ gallium,^13^ and mercury.^14^ Eutectic gallium–indium alloy (EGaIn) has emerged as one of the most viable liquid metals because of its extraordinary properties such as excellent fluidity, low melting point, high conductivity, and low toxicity, marking it a promising candidate for applications such as flexible electronics,^15^ microfluidic acoustic metamaterials,^16,17^ and conductive printing.^18^ The electrowetting of EGaIn has also been researched by controlling the surface tension of the liquid metal for active droplet manipulation.^3,18,20^ However, the electrowetting actuation of liquid metals is to date limited to the in-plane manipulation of a droplet on an electrically controlled substrate.^20−22^ Recently, liquid metal droplet jump from a surface has been explored using electrochemical surface reactions^23^ and by the contact of solid metal particles with liquid metal droplets in an electrolyte solution.^24^ Liquid metals coated with nanoparticles, called liquid metal marbles, have also been actuated electrochemically to realize opportunities for flexible liquid electronics, superstructures, and other unusual mechanical properties.^25^ However, to the best of our knowledge, the bouncing dynamics of a liquid metal droplet using electrical actuation have never been reported. Here, for the first time, we investigate the electrically induced bouncing dynamics of liquid metal droplets actuated by AC continuous electrowetting both experimentally and by simulation.

For the aqueous solution or nonmetallic liquid droplets, the jumping motion using AC electrowetting has been explored for applications in three-dimensional (3D) digital microfluidics,^26,27^ and numerical models have been developed for both jumping and bouncing processes.^28^ Various studies have been reported on the bouncing of aqueous droplets on different substrates, such as microstructured superhydrophobic surfaces without an electric field^29,30^ and topographically structured surfaces with an electric field,^31^ to analyze the impact of gravity, impact velocities, and substrate geometries. Energy-based models have also been utilized to examine the dynamical process of rebounding and adhesion of water droplets on hydrophobic surfaces.^32,33^ Moreover, the shape-dependent dynamics of a bouncing ellipsoidal drop on a superhydrophobic surface have also been investigated and reported to reduce the contact time and bouncing magnitude of droplets.^34^ It has been observed that the vertical jumping motion of aqueous solution droplets generally requires high voltage (>50 V) and can be achieved with minimal loss during the resonance.^35,36^ Thus, the droplet bouncing behavior depends on multiple factors such as the substrate roughness, hydrophobicity, liquid properties (surface tension and viscosity), ambient surrounding liquid, applied voltages, and frequencies.

The present study investigates a liquid metal (EGaIn) droplet bouncing in sodium hydroxide (NaOH) solution using continuous electrowetting for different actuation parameters, namely, the AC voltage amplitudes and frequencies. The key contributions of this study are as follows: (1) We achieve a high jump of the liquid metal droplet (∼6 mm) from a normal flat surface in a viscous solution (more viscous than air) by applying less than 10 V voltage, far lower than the previously reported voltages (50–100 V) to drive the droplet jumping from a superhydrophobic surface in the air, which is induced using an electrowetting-on-dielectric (EWOD) technique.^35,36^ (2) In addition to the experimental studies, we also simulate the liquid metal droplet bouncing and oscillation using computational fluid dynamics (CFD), to provide a systematic approach for prototyping future applications. (3) We analyze the liquid metal droplet bouncing dynamics, involving temporal changes in the center of mass of the droplet, droplet jump height, and contact length, for different actuation parameters (voltages and frequencies) of AC electrowetting. We establish that the droplet jumps higher with an increase in AC voltage amplitude and decrease in frequency, until the onset of instability. (4) We model the attenuation dynamics of bouncing cycles of the liquid metal droplet in analogy to a mechanical damped harmonic (mass–spring–damper) oscillator system.

## Materials and Methods

### Experimental Setup

**Figure 1:**
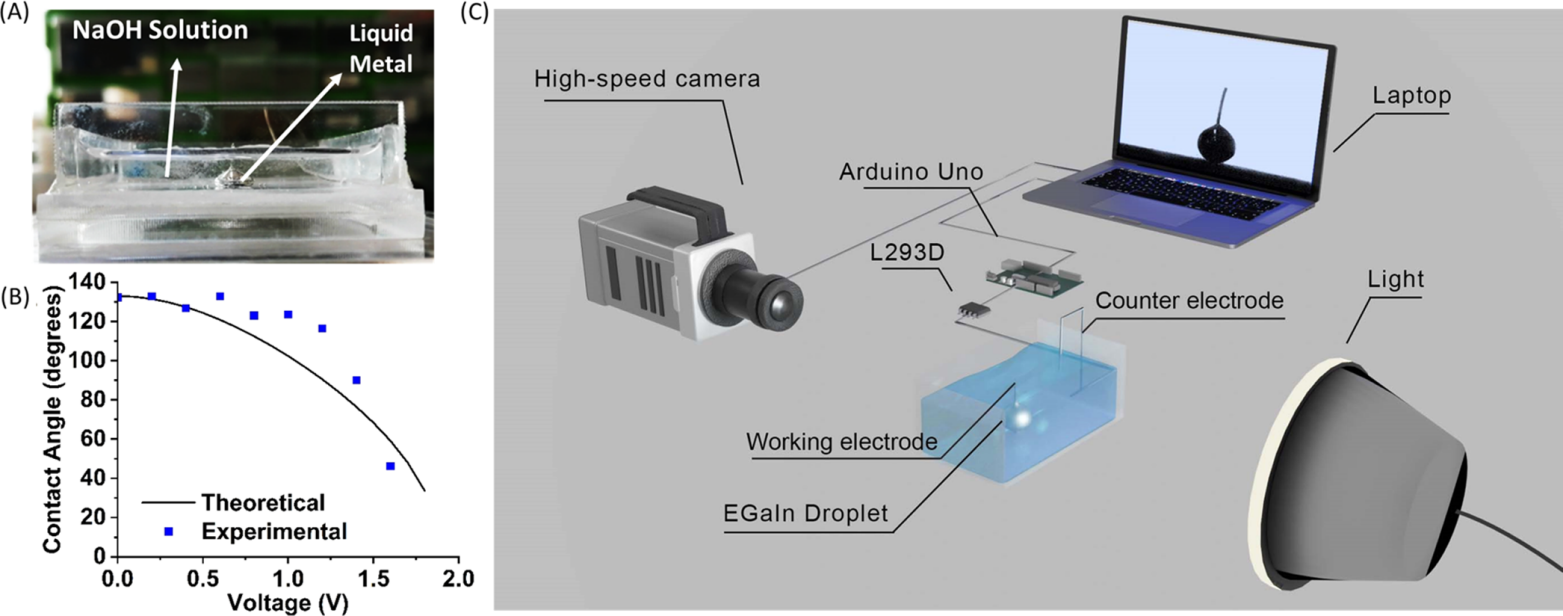
Experiments of continuous electrowetting with the EGaIn liquid metal droplet. (A) Experimental image of a liquid metal droplet in NaOH solution with wire electrodes. (B) Young–Lippmann curve plotting the change in the contact angle of the droplet with the applied DC voltage. (C) Schematic illustration of the experimental setup to record the bouncing motion of the EGaIn droplet actuated by AC continuous electrowetting.

For the experimental study, clear acrylic sheets were laser-cut and bonded with a UV-curing resin to create a rectangular bath for the NaOH solution (as shown in Figure 1A). The acrylic (PMMA) is resistant to NaOH solution, and the transparency of the bath allowed the experiment to be filmed using a high-speed camera with enough background light, as shown in Figure 1C. A small hole was cut into the base of the bed, and a silver-plated wire (diameter of 0.2 mm) was inserted as a working electrode. The electrode wire was stuck into liquid metal droplets to constantly supply current to the liquid metal during bouncing and the horizontal shift of the droplet was limited. Another silver wire electrode was positioned at the corner of the NaOH bath as a counter electrode. An Arduino Uno microcontroller board was used to generate a square wave with different frequencies. An L293D dual H-bridge motor driver was connected to the Arduino digital output, and a regulated DC power supply (Mastech HY1803D) to support a square wave for driving AC continuous electrowetting up to 600 mA of the maximum current was used.

### Choice of Materials

Eutectic gallium–indium (EGaIn) liquid metal alloy, comprising 75% gallium and 25% indium, was used for the continuous electrowetting experiments and simulations. An EGaIn droplet of 23 ± 4 μL volume (diameter of 3.6 ± 0.2 mm), 1.99 × 10^–3^ Pa·s^6^ bulk viscosity, and 509 mN/m^2,19^ surface tension was placed in a sodium hydroxide (NaOH) solution bath. NaOH (1M) was used as an electrolyte to create a basic pH environment that dissolves the surface oxide of the liquid metal,^37^ which would otherwise interfere with the wetting dynamics and contact angle.^38^ The dissolution of gallium oxide proceeds through the following reaction^39^
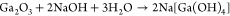
1EGaIn was preferred to other liquid metals in this study for the following reasons: (1) EGaIn is nontoxic; (2) the melting point of EGaIn (15.7 °C) is lower than standard room temperature, and so the metal remains in its liquid state, providing ease to conduct the experiments; and (3) it is highly conductive (2.94 × 10^–8^ Ω·m),^40^ having a resistivity lower than that of gallium (14 × 10^–8^ Ω·m) and mercury (90.9 × 10^–8^ Ω·m). The properties of EGaIn and other liquid metals are summarized in Table 1 for comparison.

**Table 1:** Properties of Different Liquid Metals

	mercury (Hg)	field’s metal (BiInSn)	gallium (Ga)	gallium–indium (EGaIn)
melting point (°C)	–38.83	62	29.76	15.7
toxicity	high	low	low	low
electrowetting	applicable	N/A	applicable	applicable
resistivity (Ω·m)	90.9 × 10^–8^	52 × 10^–8^	14 × 10^–8^	2.94 × 10^–8^

### Resonant Frequency

The resonant frequency (ω) of a freely oscillating liquid droplet for the *n*th mode oscillation is given as^41−43^ ω^2^ = (σ_LV_*n*(*n* – 1)(*n* + 2)/ρ)*q*^3^, where σ_LV_ is the interfacial tension of the liquid metal and surrounding electrolyte interface, *n* is the mode number, ρ is the density of EGaIn, and *q* is the characteristic length of the droplet. The resonant frequency of the actuated EGaIn droplet (*q* = 1.8 mm) is ∼55 Hz approximately.

### Experimental Procedure

For the initial DC electrowetting experiment, we used 0–2 V DC voltage to measure a change in the droplet contact angle with voltage, and the Young–Lippmann curve was plotted as shown in Figure 1B. For the AC electrowetting experiment, we applied a 4–9 V (in the step of 1 V) square wave with frequencies of 0.5, 1, 2, and 4 Hz. The experiment was conducted five times for each set of voltage and frequency. The droplet bouncing motion caused by AC electrowetting was recorded with a high-speed camera (Photron FASTCAM Nova S6 type 800K-M-32GB) at a frame rate of 2000 frames per second, as shown in Figure 1C. The contact angle was measured from the captured images using ImageJ software. The dynamic change in contact length (i.e., the base diameter of the droplet in contact with the substrate) and the height of the center of mass of the droplet from the ground surface was analyzed with our custom-made image-processing pipeline based on the OpenCV library written in Python.

## Results and Discussion

### Young–Lippmann Curve

Electrocapillarity,^44^ proposed by Lippmann,^45^ controls the surface energy of liquid metals by changing the electrical potential between electrodes in an electrolytic solution. Continuous electrowetting of liquid metal droplets is based on the same principle and electrically controls the wetting properties of a liquid–solid interface and the liquid–liquid interface.^46,47^ The movement of liquid metal droplets driven by an electric field has been modeled based on electrowetting and electrocapillarity theory.^48^ Young’s equation defines the relationship of interfacial energies with the equilibrium contact angle (θ_Y_) as^49^, where three interfacial tensions are considered between the liquid droplet and the solid substrate (σ_SL_), the solid substrate and the surrounding medium (σ_SV_), and the liquid droplet and the surrounding medium (σ_LV_).

The Lippmann equation describes the change in the effective interfacial tension (*d*σ_SL_^eff^) with the change in voltage (d*U*) as
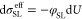
2where φ_SL_ is the surface charge density. There is no insulator between the droplet of EGaIn and the metal electrode; however, an electrical double layer (EDL) builds upon the boundary between the droplet and the surrounding sodium hydroxide solution (NaOH). The capacitance of this layer can be calculated as , where *d*_H_ is the thickness of the electrical double layer (EDL) (generally <10 nm^50−52^). Thus, for an applied voltage (*U*), the interfacial tension is given as
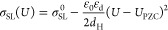
3where *U*_PZC_ is the voltage potential of zero charge and σ_SL_^0^ is the interfacial tension between the droplet and the solid substrate at zero electric field. By combining Young’s and Lippmann’s equations, we get the Young–Lippmann equation as
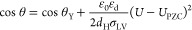
4
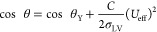
5
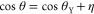
6where  is the electrowetting number,  is the capacitance per unit area, *U*_eff_ = *U* – *U*_PZC_ is the effective voltage potential, θ_Y_ is the Young equilibrium contact angle, and θ is the equilibrium apparent contact angle of electrowetting after applying voltage. The Young–Lippmann curve showing the change in the contact angle of the liquid metal droplet with the voltage is plotted in Figure 1B.

### Computational Fluid Dynamics Simulation

We used COMSOL Multiphysics 5.3 software to model the actuation of a liquid metal droplet with continuous electrowetting. We built a two-dimensional (2D) axisymmetric laminar two-phase flow model to simulate a liquid metal droplet’s deformation and bouncing motion using computational fluid dynamics (CFD). The central axis of the 2D axisymmetric model was set along the working electrode. The Navier–Stokes equation with the level set method was used to determine the liquid metal flow and measure the deforming boundary of an immiscible interface between the EGaIn and NaOH electrolyte solution. The base and outer boundaries of the NaOH solution bath were modeled as solid boundaries, and the top boundary was set as a liquid–air interface. A fine dynamic mesh (14 triangle meshes/mm) was used around the boundary of the liquid metal droplet to precisely model the dynamical liquid metal surface movement, while a coarser mesh (11 triangle meshes/mm) was used toward the more static region of the NaOH solution. A total number of 16,379 mesh elements were used in the entire simulation domain (5 mm × 10 mm).

**Figure 2:**
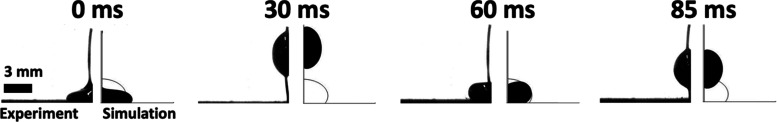
Image instants for four different time frames during electrically induced bouncing of the liquid metal droplet by both experiments and simulations. At each captured time frame, the (left-half) experimental and (right-half) simulated half droplet images are shown in the left and right quadrant in parallel, respectively. A 23 μL EGaIn droplet was actuated using AC electrowetting with a 5 V square wave at a frequency of 0.5 Hz. The images were captured at time frames when the first and second maximum deformations and the maximum jump height were achieved [Supporting Information Videos].

We first examined the accuracy of our electrowetting actuation model for an EGaIn droplet (23 μL) with multiple DC voltages. We supplied the DC voltage to the working electrode wire in the range from 0 to 2 volts with a step of 0.2 V, where 0.2 s intervals were set to allow enough time for the EGaIn droplet to deform. We measured the contact angle of the liquid metal droplet at each voltage and plotted the Young–Lippmann curve (Figure 1B). We observed that the simulation model and experimental values show a good agreement. Utilizing the same electrowetting actuation model and experimental setup, we examined the EGaIn droplet bouncing by applying AC square voltages with multiple amplitudes and frequencies in both simulations and experiments. We investigated the voltage range from 4 to 9 V with a step of 1 V. Figure 2 shows the captured images of the bouncing droplet at the maximum stretch states and the maximum jump states, when a 23 μL EGaIn droplet was actuated by a 5 V square wave with a frequency of 0.5 Hz. Experimental results (left-half droplet) and simulation model outputs (right-half droplet) for each time frame show good agreement for the electrically induced bouncing motion of the droplet.

### Droplet Bouncing Dynamics: Experiments and Simulations

**Figure 3:**
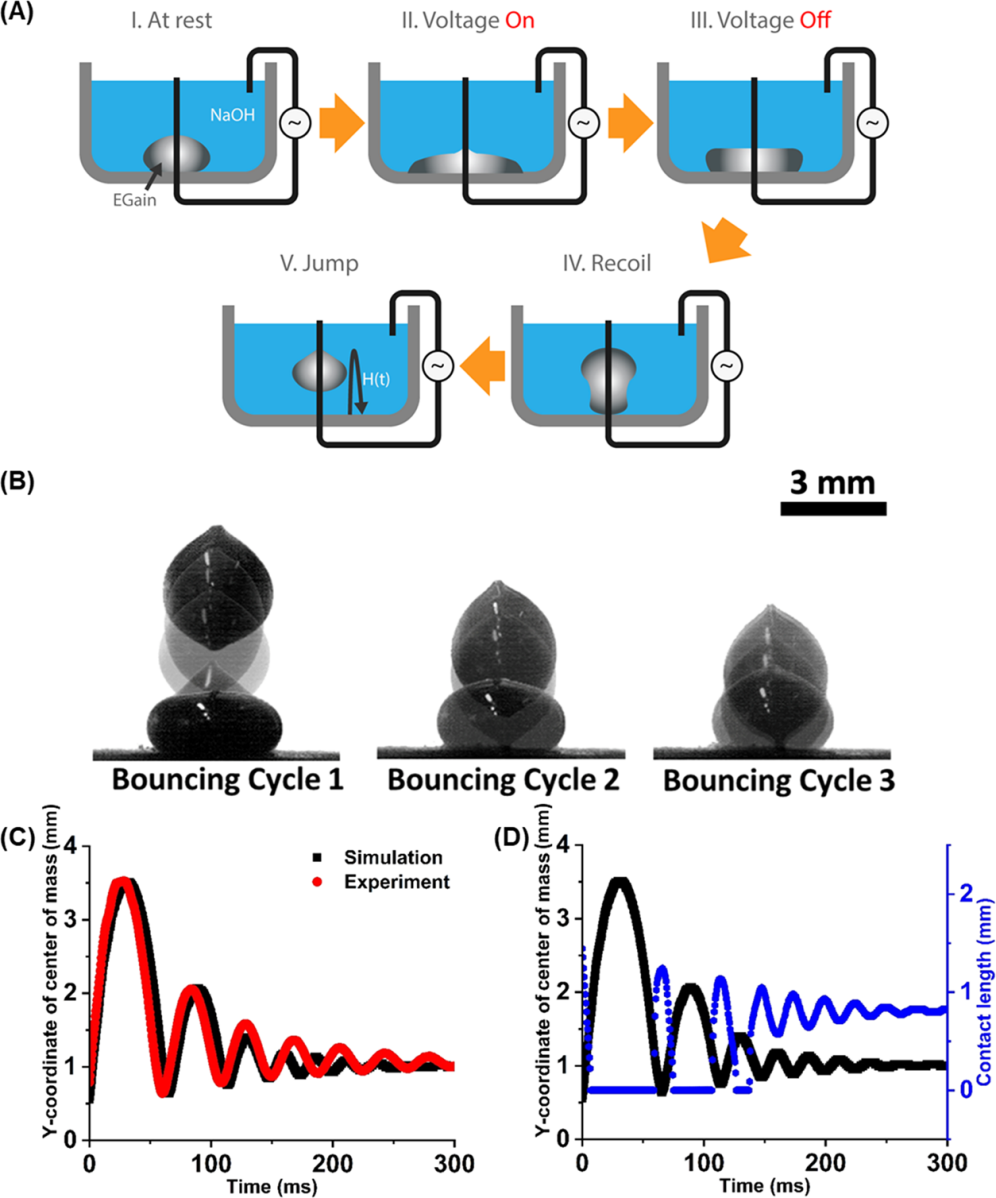
Electrically induced bouncing dynamics of the droplet. (A) Process state transition diagram of actuated liquid metal: (I) Initial unactuated state, (II) spread state when the voltage is applied, (III) retract state when the voltage drops to zero, (IV) recoil, and (V) jump. (B) Superimposed experimental images of the droplet during different bouncing cycles upon actuation with 5 V voltage at 0.5 Hz frequency. (C) Time evolution of the average height (mm) of the center of mass of a bouncing droplet. The red line shows an experimental result, and the black line shows a simulation output when a 5 V square wave is applied with a frequency of 0.5 Hz. (D) Complementary relationship of the height (mm) of the center of mass (black) and the contact length of the droplet (blue) from the simulation (i.e., when one increases, the other decreases).

The electrically induced bouncing process can be classified into five stages as shown in Figure 3A: (I) an EGaIn droplet is at rest, initial unactuated state; (II) the droplet spreads when a positive voltage is applied to the droplet; (III) retraction occurs when the applied voltage drops to zero because of the increase in interfacial tension; (IV) the droplet recoils along the working electrode as the droplet momentum shifts from the horizontal axis to the vertical axis; and (V) the droplet is detached from the surface with an excessive amount of momentum^27^ and bounces in the electrolyte solution until the momentum drops to zero due to the force of the gravity.

We captured different bouncing cycles of the liquid metal droplet with a high-speed camera, and a few superimposed droplet images for electrowetting actuation with a 5 V square wave of 0.5 Hz frequency are shown in Figure 3B. To track the time evolution of the bouncing motion, we captured the trajectory of the center of mass of the liquid metal droplet using the OpenCV-Python image-processing pipeline. We recorded the height of the center of mass and contact length of the droplet on the ground plane by detecting the droplet as a blob in a binary-processed image. The experimental result (red line) and the simulation model (black line) show good agreement, as shown in Figure 3C.

We also measured the contact length of the droplet and found that it increases with the decrease in the height of the center of mass (i.e., the jump height), as shown in Figure 3D. This result supports the complementary relationship of the jump height and contact length, as a liquid metal droplet conserves the momentum in horizontal spreading motion and vertical jumping motion.

**Figure 4:**
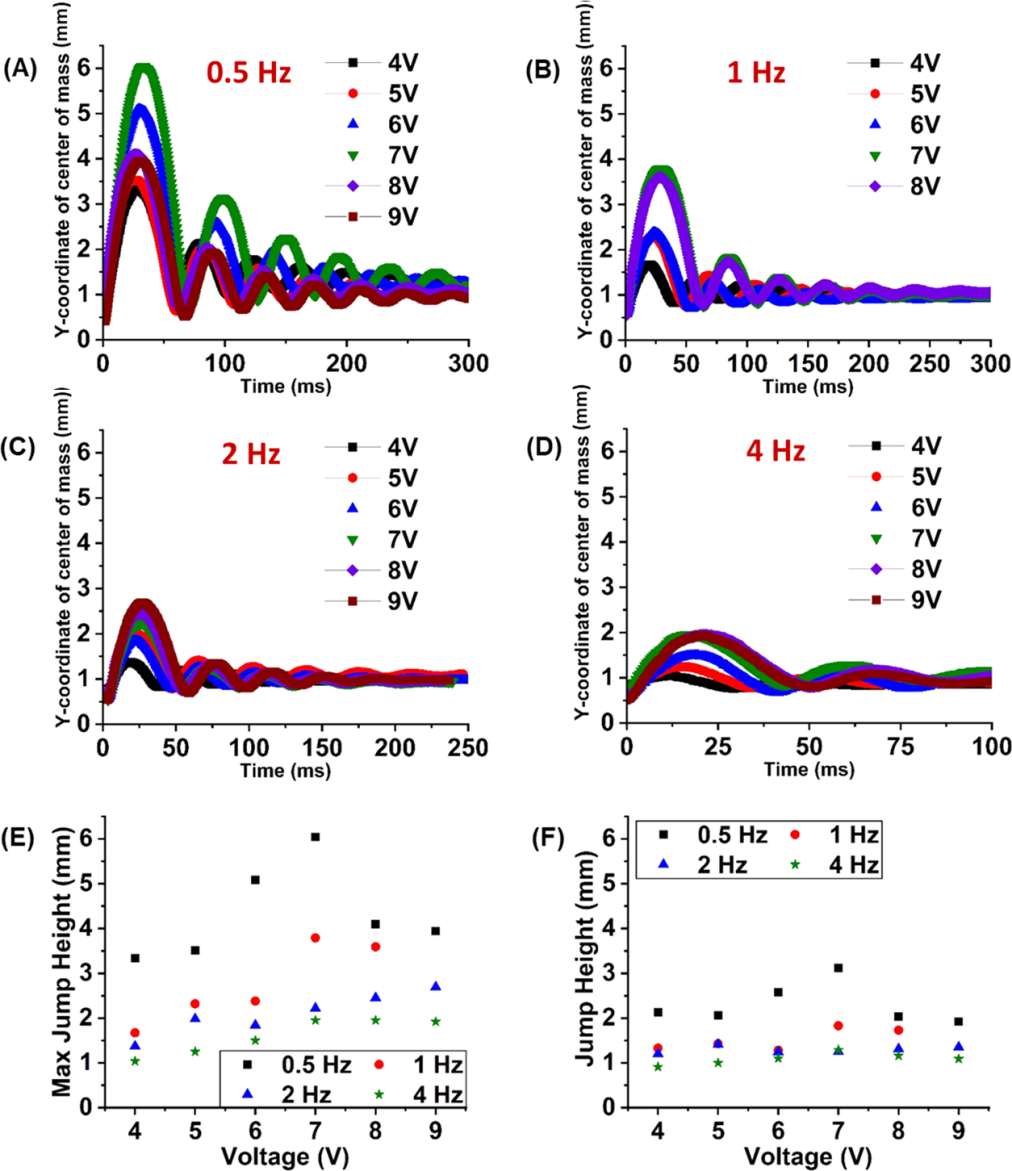
Bouncing trajectories of the center of mass of the EGaIn droplet by CEW actuation at different voltages and frequencies. (A–D) Temporal behavior of the center of mass of the droplet launched with a square wave of different applied voltages (4–9 V in the step of 1 V) at frequencies of 0.5, 1, 2 and 4 Hz, respectively. (E, F) Maximum jump height for (E) first bouncing cycle and (F) second bouncing cycle of the liquid metal droplet upon actuation with the CEW at different voltages and frequencies.

We empirically studied the droplet bouncing dynamics actuated by AC electrowetting with a square wave voltage ranging from 4 to 9 V with a step of 1 V and frequency of 0.5, 1, 2, and 4 Hz. The time evolution of the height of the center of mass of the droplet for different voltages is plotted in Figure 4A–D. For every frequency, we observed that the maximum height of the droplet in the first bouncing cycle increased as the applied voltage was increased. This is because an increase in the applied voltage leads to a more spread droplet area with a larger droplet contact length. Thus, when the applied voltage drops to zero, the droplet gains more kinetic energy as it retracts from the more spread state due to the higher voltage input. Since the kinetic energy of the droplet is converted into the gravitational potential energy in the recoiling stage, a larger jump height is achieved by a higher voltage. However, we observed that the jump height decreased above 7 V for some frequencies. This is attributed to an instability causing satellite droplets and electrolysis bubbles around the surface of the droplet for electrowetting at above 7 V (i.e., 8 and 9 V input voltages). Faradaic reactions are caused at the boundary of liquid metal/electrodes and NaOH electrolyte at high electrowetting voltages, which causes electrolysis and generates bubbles of oxygen or hydrogen depending on whether the potential applied to the liquid metal is positive (oxidizing) or negative (reducing), respectively.^39^

The partial equations for electrolysis of water are as follows:

(a) At the anode (positive electrode), the oxygen anions are oxidized when they lose electrons: 2H_2_O → O_2_ + 4H^+^ + 4e^–^.
(b) At the cathode (negative electrode), the hydrogen cations are reduced when they gain electrons: 2H_2_O + 2e^–^ → H_2_ + 2OH^–^.

Hence, the net reaction of the electrolysis is 2H_2_O → 2H_2_ + O_2_.

The instability reduces the kinetic energy of the liquid metal droplet and lowers the maximum jump height, as shown in Figure 4E,F. The maximum height observed in these experiments is 6 mm, which is less than the theoretical value of 14.6 mm reported in the literature,^23^ due to the unaccounted system losses such as viscous drag and viscoelastic dissipation.

The driving frequency also affected the maximum height of the droplet center of mass and the following bouncing heights, as shown in Figure 4. We observed that as the applied frequency increased from 0.5 to 4.0 Hz, both the maximum jump height and subsequent bouncing height decreased. This is because as the frequency of electrowetting actuation increases, the time available for the droplet to spread on the ground decreases, and consequently, the droplet gains less kinetic energy to jump.

### Effect of Droplet Size

With the applied voltage, the base radius also increases until saturation, thereby leading to an increase in the surface area (Δ*A*). The excess interfacial surface energy (Δ*E*_S_) is related to the potential energy (Δ*E*_P_) as σ_LV_ Δ*A* = ρ*ghV*_l_ where *g* is the gravitational constant, *V*_l_ is the droplet volume, and *h* is the jumping height.^23^ Thus, the ratio of change in the surface area (Δ*A*) to the droplet volume (*V*_l_) affects the droplet bouncing dynamics. The droplet bouncing through droplet coalescence or impact collisions is promoted by increasing the droplet diameter but suppressed by decreasing it.^53^ In these cases, by increasing the droplet diameter, the Ohnesorge number, i.e., the ratio of viscous forces to the inertia and surface tension forces, decreases and enhances the bouncing dynamics.^31,54^

### Energy Cycle

**Figure 5:**
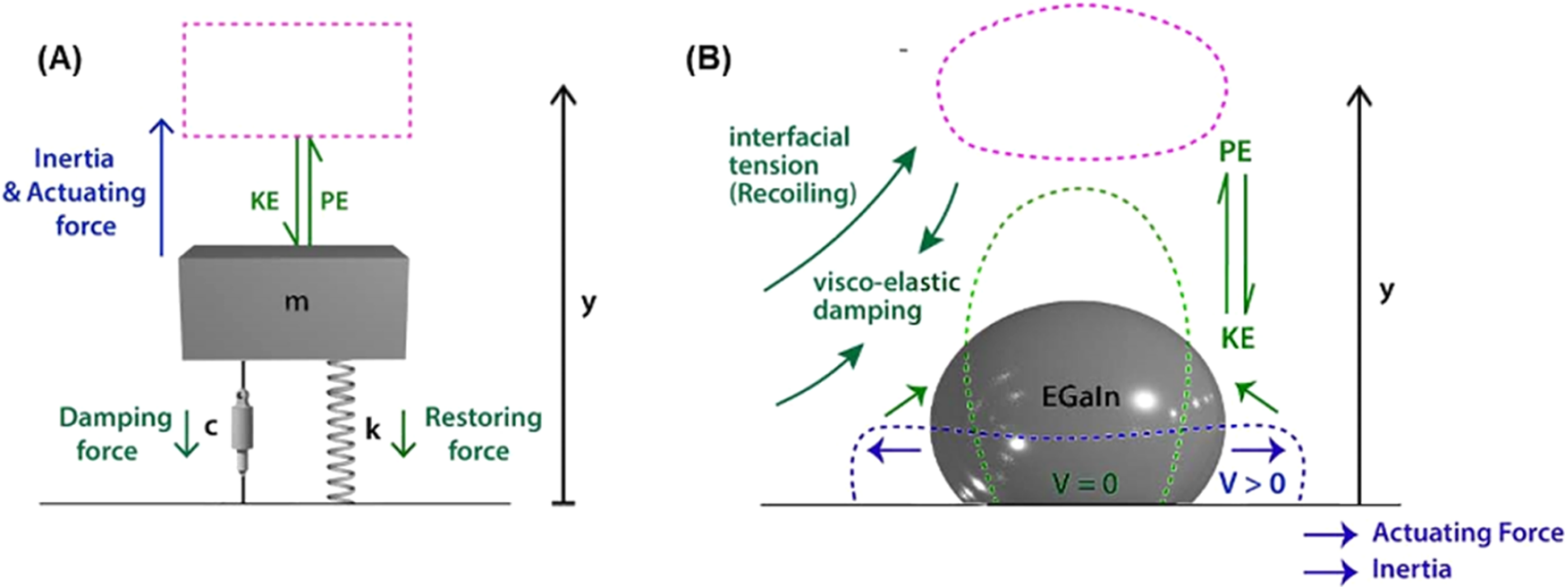
Schematic showing mechanical analogy of (A) simple damped harmonic oscillator (mass–spring–damper system) and (B) liquid metal underdamped droplet bouncing system.

The droplet bouncing process involves multiple energy conversion cycles, as shown in Figure 5. First, the interfacial tension of the liquid metal–electrolyte interface decreases due to the applied electric field. This decrease in interfacial tension causes the droplet to spread to balance the interfacial energies (*E*_s_). The surface energy is given as
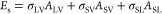
7where σ_LV_, σ_SL_, and σ_SV_ are the three interfacial tensions and *A*_LV_, *A*_SL_, and *A*_SV_ are the surface areas for the liquid droplet and the surrounding medium interface, the liquid droplet and the solid substrate interface, the solid substrate and the surrounding medium interface, respectively.

Subsequently, when the electric field (and electrowetting force) is removed, the interfacial tension increases and retracts the droplet to its initial minimum surface energy state. In the retraction stage, the surface energy stored during electrowetting is converted into kinetic energy (Δ*E*_K_), stated as Δ*E*_K_ = 1/2 ρ*V*_l_ (2π*fa*)^2^, where ρ is the density of EGaIn, *f* is the actuation frequency, a is the amplitude of droplet motion, (2π*fa*) is the speed of the moving EGaIn droplet, and *V*_l_ is the volume of the droplet (23 μL).

When the total energy of the droplet, i.e., the sum of instantaneous surface energy and kinetic energy, exceeds the initial surface energy of the droplet, the excess amount of energy makes the droplet jump. Subsequently, assuming that the excess interfacial surface energy (Δ*E*_S_) is entirely converted into the gravitational potential energy (Δ*E*_P_ = ρ*ghV*_l_) and causes the droplet to bounce off the ground surface, we get σ_LV_ Δ*A* = ρ*ghV*_l_, where *g* is the gravitational constant, Δ*A* is the difference in the surface area of the liquid metal at the spread state and the unactuated state, and *h* is the jumping height. The upper limit of jumping height using this expression was determined to be 14.6 mm.^23^ The bouncing motion attenuates because of the energy loss incurred by the viscoelastic damping force, drag force from the surrounding medium, and inelastic collisions of the droplet on the substrate. The liquid metal droplet stabilizes to the equilibrium position after a few bouncing cycles. Then, it restarts from the initial unactuated electrowetting stage and repeats the same energy cycle to attain a bouncing motion. The dynamical change in interfacial tension that determines the bouncing behavior of a liquid metal droplet can be controlled by the magnitude and frequency of the applied voltage through AC continuous electrowetting.

### Mass–Spring–Damper Model

We model the attenuation dynamics of a series of underdamped droplet bouncing cycles in analogy to a simple damped harmonic oscillator (mass–spring–damper model^55^), as shown in Figure 5. The droplet bouncing system is driven by the imbalance of interfacial tension energies, droplet kinetic energies, and potential energies after the electrowetting force is removed (i.e., the actuation voltage drops to 0 V). The spring–mass–damper model used to fit the bouncing cycles also depends on the actuation parameters, which lead to different bouncing heights of the droplet, contact lengths, and contact time durations. A general bouncing motion of a liquid metal droplet can be classified into two motion regimes:^55^ free flight motion and underdamped oscillation motion.

If the initial jump height driven by electrowetting is high enough, the liquid metal droplet lifts off the substrate and follows free flight motion where the contact length becomes zero as shown in Figure 3D.

After colliding the substrate (contact length > 0), the liquid metal droplet undergoes underdamped bouncing motion till the center of mass of the liquid metal stabilizes to the equilibrium position where the gravity force on the liquid metal is balanced by the interfacial tension of the liquid metal and the substrate.

We applied a piecewise mass–spring–damper model^55^ to realize both free flight motions and underdamped oscillation motions of bouncing droplets based on the contact state with the substrate [details in the Supporting Information]. The trajectory of the *i*th bouncing cycle, which consists of the free flight motion (*y*_1*i*_(*t*)) and the underdamped oscillation motion (*y*_2*i*_(*t*)), is expressed based on the condition of liquid metal contact length (*L*) at the substrate as follows
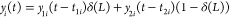
8where

 and *t*_1*i*_ is the time when the liquid metal is detached from the substrate and *t*_2*i*_ is the time when the liquid metal hits the substrate in the *i*th bouncing cycle.

**Figure 6:**
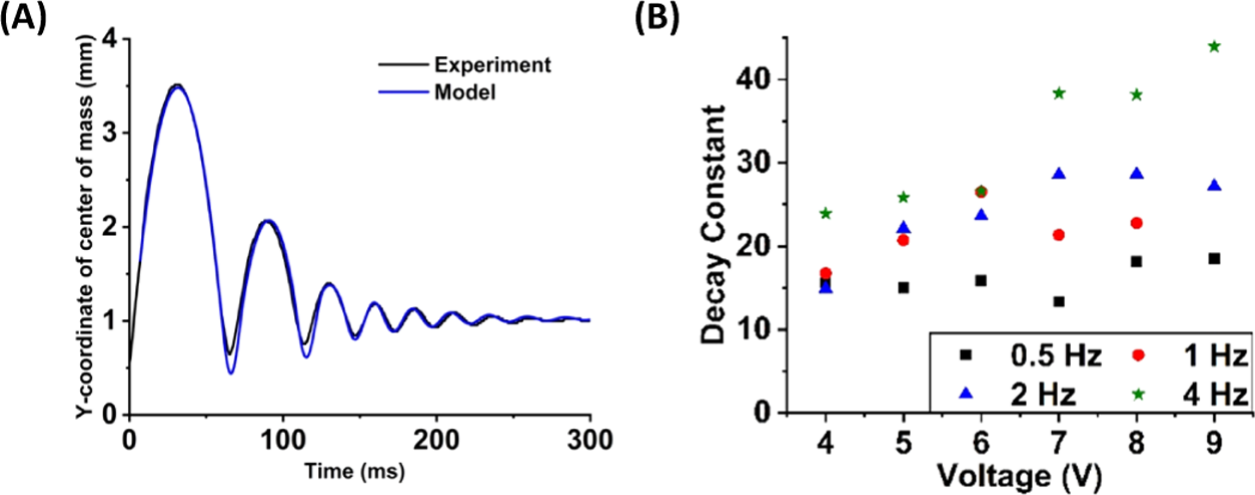
(A) Bouncing motion of the liquid metal droplet by electrowetting actuation at 5 V and 0.5 Hz frequency. The black curve plots the experimental data, and the blue curve plots the piecewise bouncing model. (B) Decay constant of the underdamped droplet bouncing cycles by electrowetting at different voltages and frequencies.

The model fits the bouncing dynamics of the experimental data, as shown for the case when the liquid metal was actuated by 5 V voltage at 0.5 Hz frequency in Figure 6A. The initial conditions of the height and speed of *y*_1*i*_ (*t*) and *y*_2*i*_ (*t*) in the bouncing cycles were calculated from the continuity of the free flight motion and the underdamped oscillation motion at the transition time (i.e., *t*_1*i*_ and *t*_2*i*_). The initial speed and the height of the flight motion of the first cycle were given by the experimental data at the time *t*_11_ = 7 ms, when the liquid metal was observed to lift off the substrate after the release of the electrowetting force (i.e., contact length becomes zero for the first time in Figure 3D). The transition time of each cycle was measured from the experimental data when the contact length (*L*) is switched between zero and positive values in Figure 3D.

The root-mean-square deviation (RMSD) of the model from the experimental result is 0.05 mm.

Since the first and second bouncing cycles in Figure 6A (i.e., 7 ≤ *t* < 126.5 ms) include the high free flight motion, the large momentum of the falling liquid metal leads to the deviation of the underdamped oscillation motion model from the experimental result with the RMSD of 0.08 mm. From the third bouncing cycle (i.e., *t* ≥ 126.5 ms), the impact of the free flight motion on the underdamped oscillation motion becomes negligible due to the decay of the jump height, and the mass–spring–damper model accurately expresses the attenuation of the bounce until the liquid metal stabilizes to the equilibrium position with the RMSD of 0.02 mm.

With a simplified mass–spring–damper model, the underdamped bouncing of the liquid metal droplet can be expressed by *mÿ* + *cẏ* + *ky* = *mg*, where *k* is a spring constant, *c* is a damping coefficient, *m* is a mass of the liquid metal droplet, *g* is the gravitational constant, and *y* is the displacement of the center of mass of the liquid metal from the substrate. The damping coefficient (*c*) is equivalent to the viscous damping coefficient of the moving EGaIn droplet in the NaOH electrolyte solution, and the spring constant (*k*) is comparable to the interfacial tension between the substrate and liquid metal (σ_LV_ ∼ *k*).

An exponentially decaying solution for the underdamped harmonic oscillator equation is given as follows^56^
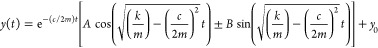
9where *y*_0_ is the equilibrium position when the liquid metal droplet remains at rest without electrowetting (i.e., *ky*_0_ = *mg*).

We determined the peak height values (*y*_p_) of each bouncing cycle from the plotted trajectories of the center of mass of the bouncing droplet (Figure 4). We fit the measured peak values in an exponential curve equation, *y*_p_(*t*) = α exp(−β*t*) + *y*_0_, to determine the decay constant (β) of the underdamped harmonic oscillator model.^57^ The power of the fitted curve (β) is expressed by *c*/2*m* as shown in the equation given above. We calculated the decay constants of the system for different voltages and frequencies to analyze the attenuation dynamics in a series of bouncing cycles. The decay constant of the underdamped bouncing cycles increases as the amplitude of the applied AC voltage and its frequency increases, as shown in Figure 6B. This implies that the bouncing motion fades faster with larger voltage and frequency.

## Conclusions

Dynamical actuation of high-density liquid metals against gravity can revolutionize multiple sectors with applications ranging from 3D microfluidics to energy harvesting. While the vertical bouncing motion of nonmetallic droplets has been well investigated by the AC electrowetting actuation method, the same phenomenon has rarely been reported for liquid metal droplets. Here, we investigated the electrically induced bouncing motion of a liquid metal droplet using less than 10 V voltage and low-frequency AC continuous electrowetting. We explored the bouncing dynamics of eutectic gallium–indium alloy (EGaIn) droplets for different driving frequencies and voltages of an AC square wave. We achieved droplet bouncing at voltages (<10 V) and frequencies (≤4 Hz) that are far lower than the resonant frequency of the droplet (55 Hz). AC continuous electrowetting actuation allows a droplet to bounce from a non-superhydrophobic surface in more viscous medium than the air. We investigated the droplet bouncing dynamics by both the CEW experiments and CFD simulations and analyzed the effect of the applied AC voltage amplitudes and frequencies on the droplet bouncing trajectories. The maximum droplet jump height was found to increase with an increase in voltage and decrease in frequency until we observed an instability such as the formation of satellite droplets or electrolysis. The bouncing motion tends to fade out slowly because the energy is lost by the inelastic collision of the droplet with the substrate and by viscous drag through the electrolyte solution. We explained the electrically induced droplet bouncing motion based on energy conversion cycles and modeled the attenuation of consecutive bouncing cycles using a mass–spring–damper model. Our experimental results show that the decay constant of the underdamped bouncing cycles increases with an increase in voltage and increase in frequency. This study about the electrical control of liquid metal droplet bouncing using a facile AC electrowetting technique can open a plethora of applications such as 3D digital microfluidics,^58^ RF switching,^1^ energy harvesting,^59^ portable lab-on-a-chip applications,^60^ and heat transfer systems.^61^

## Supporting Information Available

Droplet bouncing model, including free flight motion and underdamped oscillation motion (PDF)
Simulated video of an EGaIn droplet (23 μL) actuated using AC electrowetting with a 8 V square wave at a frequency of 1 Hz (MP4)
Experimental recording of an EGaIn droplet (23 μL) actuated using AC electrowetting with a 5 V square wave at a frequency of 1 Hz (MP4)
Experimental recording of an EGaIn droplet (23 μL) actuated using AC electrowetting with a 5 V square wave at a frequency of 0.5 Hz (MP4)
Simulated video of an EGaIn droplet (23 μL) actuated using AC electrowetting with a 5 V square wave at a frequency of 0.5 Hz (MP4)
Short summary video of the work (MP4)
