# Supplementary material for: Electrically Induced Liquid Metal Droplet Bouncing
Source: Langmuir. 2022 May 26;38(22):6996–7004. doi: 10.1021/acs.langmuir.2c00577 (PMC9178922; doi:10.1021/acs.langmuir.2c00577)
Supplement: Supplementary file 1 — la2c00577_si_001.pdf [file la2c00577_si_001.pdf]

Supplementary Information

Electrically Induced Liquid Metal Droplet Bouncing

Shubhi Bansal[1], Yutaka Tokuda[2], Jonathon Peasley[3] and Sriram Subramanian[1]*

[1] University College London, London WC1E 6BT, UK
[2] City University of Hong Kong, Kowloon, Hong Kong, China
[3] University of Sussex, Brighton, UK

e-mail: shubhi.bansal@ucl.ac.uk

Table of contents:

1. Droplet bouncing model
   A. free flight motion
   B. underdamped oscillation motion

1. Droplet bouncing model

A general bouncing motion of a liquid metal droplet can be classified into two motion regimes[1]: free flight motion and underdamped oscillation motion.

A. Free flight motion (contact length = 0)

When a liquid metal (LM) droplet is detached from the substrate (i.e. contact length is zero), it follows the free-flight motion in the NaOH bath. The forces applied to an LM include gravitational force, buoyant force and the viscous drag force by Stokes's law. We can express the equation of the LM in free-flight motion by Newton's second law as-

$$m\ddot{y} + (6\pi\mu r)\dot{y} = mg - V_l\rho_e g \quad (1)$$

where y is the height of the centre of the mass of LM from the substrate, m is the mass of LM, μ is the dynamic viscosity of NaOH solution, r is the radius of LM, V_l is the volume of LM, ρ_e is the density of electrolytic NaOH solution, and g is the gravitational constant. We can analytically solve the Eq. (1) as-

$$y_1(t) = v_t t + v_0\tau\left[1 - e^{-t/\tau}\right] + v_t\tau\left[e^{-t/\tau} - 1\right] \quad (2)$$

where v_t is the terminal velocity of the falling LM, v_0 is the initial speed of LM when it is detached from the ground, and τ is the characteristic time constant ($\tau = m/6\pi\mu r$). From the Eq. (1), the terminal velocity v_t is calculated as

$$v_t = \dot{y}_1(t = \infty) = \frac{(m - V_l \rho_e)g}{6\pi\mu r} \quad (3)$$

B. Underdamped oscillation motion (contact length > 0)

When the LM droplet is in contact with the substrate, the forces applied to LM include the damping force and the spring force of LM. The LM motion can be expressed by a mass-spring-damper model as-

$$m\ddot{y} + c\dot{y} + ky = mg \quad (4)$$

where c is the damping coefficient, and k is the spring constant. The solution of underdamped harmonic oscillation in Eq. (4) is given as-

$$y_2(t) = e^{-(c/2m)t}\left[A\cos\left(\sqrt{\left(\frac{k}{m}\right) - \left(\frac{c}{2m}\right)^2}\,t\right) + B\sin\left(\sqrt{\left(\frac{k}{m}\right) - \left(\frac{c}{2m}\right)^2}\,t\right)\right] + y_0 \quad (5)$$

where y_0 is the equilibrium position when the LM droplet remains at rest without electrowetting (i.e., $ky_0 = mg$).

References-

(1) Mark, N.; Shuguang, H. A Mass-Spring-Damper Model of a Bouncing Ball. *Proc. 2004 American Control Conf. ACC* 2004, **2004**, *1*, 499-504. https://doi.org/10.23919/ACC.2004.1383652.
